# Probe‐based confocal laser endomicroscopy for pleural malignancies diagnosis

**DOI:** 10.1111/resp.13945

**Published:** 2020-10-01

**Authors:** Olivier Bonhomme, Vincent Heinen, Nancy Detrembleur, Jean‐Louis Corhay, Renaud Louis, Bernard Duysinx

**Affiliations:** ^1^ Pneumology Department CHU Liège, Domaine Universitaire du Sart‐Tilman Liège Belgium; ^2^ Pathology Department CHU Liège, Domaine Universitaire du Sart‐Tilman Liège Belgium

**Keywords:** medical thoracoscopy, pleural carcinomatosis, pleuroscopy, probe‐based confocal laser endomicroscopy

## Abstract

**Background and objective:**

Probe based confocal laser endomicroscopy (pCLE) is an optical imaging technique allowing live tissue imaging at a cellular level. Currently, this tool remains experimental. Two studies regarding pleural disease have been published and suggest that pCLE could be valuable for pleural disease investigations. However, normal and malignant pleural pCLE features remain unknown. Therefore, we conducted a prospective trial of pCLE during medical thoracoscopy to study and describe the malignant and benign pleural pCLE features.

**Methods:**

Every patient >18 years referred to our department for medical thoracoscopy was eligible. Medical thoracoscopy was performed under sedation, allowing spontaneous breathing. Five millilitres of fluorescein (10%) was intravenously administrated 5 min before image acquisition. The pCLE was introduced through the working channel of the thoracoscope and gently placed on the parietal pleura to record videos. Afterwards, biopsies were performed on the corresponding sites. Malignant and benign pleural pCLE features were precisely described and compared using 11 preselected criteria.

**Results:**

A total of 62 patients were included in the analysis including 36 benign and 26 malignant pleura. Among our preselected criteria, ‘abnormal tissue architecture’ and ‘dysplastic vessels’ were strongly associated with malignancies (100% and 85% ss, 721% and 74% sp, respectively) whereas, the ‘full chia seeds sign’ and ‘cell shape homogeneity’ were associated with benignity (36% and 56% ss, 100% and 70% sp, respectively). No study‐related adverse events occurred.

**Conclusion:**

Benign and malignant pleural involvement have clearly distinct pCLE features.

## Abstract 2

Probe based confocal laser endomicroscopy (pCLE) is an optical imaging tool allowing live imaging of tissues at a cellular level. It remains experimental but its clinical value as a diagnostic/guiding tool is apparent. To address the lack of data in thoracic oncology and pleural diseases, we show the ability of pCLE during medical thoracoscopy to distinguish benign from malignant pleural involvement.

**See related** Editorial

**Abbreviations**
NPV: negative predictive value
pCLE: probe‐based confocal laser endomicroscopy
PPV: positive predictive value
sp: specificity
ss: sensitivity

## INTRODUCTION

Confocal laser endomicroscopy is an optical endoscopic imaging tool.^1^ This technique allows live in vivo imaging of tissues at a cellular level with a video frame of 12 images per second. In probe‐based confocal laser endomicroscopy (pCLE), the device is miniaturized so that it can be introduced through the working channel of endoscopes providing live, dynamic and microscopic assessment of tissues.

pCLE remains an experimental technique and data are scarce in pulmonary medicine and thoracic oncology.^2^, ^3^, ^4^, ^5^, ^6^, ^7^, ^8^, ^9^ As for pleural diseases, Zirlik *et al*. showed that, compared to pleural fluid cytology, pCLE can detect malignant cells in pleural effusion with 87% sensitivity and 99% specificity.^9^ Wijmans *et al*. have recently published a prospective (*n* = 15) study assessing (p)CLE for guiding biopsies in the specific indication of suspected malignant pleural mesothelioma with different sampling methods. They showed that (p)CLE could distinguish malignant mesothelioma from pleural fibrosis with high precision.^8^ However, only three thoracoscopies, which is the gold standard procedure for malignant mesothelioma management,^10^, ^11^, ^12^ were included in that study. Otherwise, we have recently reported on three cases of pCLE during medical thoracoscopy with the first description of the parietal pleura during pCLE in a healthy patient and two patients with malignant pleural involvement.^13^

Although these results suggest that pCLE could be valuable for pleural diseases investigation, large studies describing pleural pCLE in healthy subjects or in pathological conditions are still lacking and needed. Consequently, we conducted a prospective study to assess the pleural pCLE features obtained during thoracoscopy. The main objectives were to confirm our previous descriptions over a larger population, to assess the feasibility and safety of the technique and to identify specific criteria for benign or malignant pleural involvement.

## METHODS

### Study design and procedure

We performed a single‐centre prospective cross sectional study. Every patient ≥18 years referred for a medical thoracoscopy between June 2018 and September 2019 (pleural effusion work up, recurrent pneumothorax, talc pleurodesis, etc.) was eligible for the present study. Exclusion criteria were pregnancy and known allergy to fluorescein. Thoracoscopy was performed under sedation (allowing spontaneous breathing) by two experienced investigators with a systematic procedure. The patient was positioned in lateral decubitus during the intervention. After chest ultrasound examination, a pneumothorax was induced with the Boutin trocar and one entry port was performed through the chest wall. Two different thoracoscopes were used during this study: Wolf thoracoscope with an outer diameter of 7 mm (Richard Wolf GmbH, Knittlingen, Germany) and a Storz single puncture thoracoscope with an outer diameter of 10 mm (Karl Storz GmbH, Tuttlingen, Germany). Five millilitres of fluorescein (10%) was intravenously administrated 5 min before image acquisition. Thereafter, the pleural cavity was examined macroscopically. Then, the pCLE (Alveoflex, lateral resolution 3 μm, optical area 1.13 mm^2^, depth of focus 0–50 μm) (Cellvizio; Maunakea Technologies Paris, France) was gently placed on the parietal pleura and videos were recorded. If macroscopic abnormalities were noticed, pCLE was performed on the affected zones. In the absence of macroscopic abnormality, three random sites were selected. Finally, biopsies were systematically performed on the same sites. During the procedure, the thoracoscopists rated the quality of the pCLE acquisitions as good, acceptable or low.

We previously reported our first experience of pCLE during medical thoracoscopy.^13^ For this publication, we performed pCLE in seven patients but only three were reported. This was the first step of our research. Afterwards, in collaboration with the pathologists from our centre, the pCLE acquisitions and the histological sections of the seven patients were reassessed. The objective was to identify the potential discriminant criteria between benign and malignant pleura. Eleven criteria were selected (Table 1, Appendix S1, Fig. S1 in Supplementary Information) and were prospectively assessed in the present study which is the second step of our research. No training session was performed after the first step. The seven patients of step 1 are not included in the present study to avoid the risk of bias. After video acquisition, a third blinded investigator screened every video set to select the five best representative images per patient for further analysis and assessment of the preselected criteria.

**Table 1:** Criteria for pCLE pleural evaluation

Criteria	Details
Abnormal tissue architecture	**No:** Correct identification of the previously described normal pleura characteristics^†^ Mesothelial monolayer with a regular distribution of the cells Fibroadipose connective tissue with regular and homogeneous vascularization Muscular fibres
	**Yes:** Identification of cellular/tissue structures which are not known to belong to the normal pleura (cellular clusters or dark clumps, glands, cells cordons, dysmorphic cells, papillar distribution, etc.)
	**Not assessable**
Cellular homogeneity in size, shape and fluorescence	Subjective description by the investigator with reference to the normal pleura^†^
	**Yes**
	**No**
	**Not assessable**
Mean cellular size	Mean cellular size assessed on a full optical area (1.13 mm^2^)
	**Not assessable**
Mean cellular density (number of cells/10.000 μm^2^)	Mean of three measurements realized in areas where the cellular density was visually the highest
	**Not assessable**
Dysplastic vessels^‡^	**Yes** ^**§**^: Presence of tortuous vessels Vascular leaks of fluorescein Giant vessels
	**No:** No criteria for dysplastic vessel
	**Not assessable**
Max vascular diameter^‡^	Selection and measurement of the largest vascular diameter on the set of images
	**Not assessable**
Max vascular density^‡^	Number of assessable vessels/optical area (1.13 mm^2^)
	**Not assessable**
Connective tissue fibres organization	**Anarchic:** Coarse fibres, irregular in shape or direction, without well‐defined architecture
	**Organized:** Regular in shape and direction, well‐defined architecture
	**Not assessable**
Full chia seed sign	**Yes:** Full optical area with chia seed sign (Fig. 1C)
	**No:** Absence or presence on only one portion of the optical area
	**Not assessable**

^†^ Normal pleural pCLE description as previously reported by Bonhomme *et al*.^13^

^‡^ Vessels are defined as continuous tubular hyperfluorescent images of at least 50 μm.

^§^ As described by Cannizzaro *et al*.^20^

pCLE, probe‐based confocal laser endomicroscopy.

The criteria were not scored if assessable elements for their interpretation were missing. Our study was approved by the CHU Liege ethics committee (B707201837069). Every patient provided written informed consent.

### Statistical analysis

The Fisher's exact test was used to analyse the link between pCLE qualitative variables and the final histological diagnosis. For quantitative variables, the unpaired t‐test was used. If a criteria was not assessable for a patient during the investigation, it was excluded from the statistical analysis.

## RESULTS

### Patients' characteristics, feasibility and safety

A total of 64 patients (44 males and 20 females) were recruited. Two patients were excluded: pCLE images no more available for one patient and other one because of a lack of histological conclusion. Of the 62 remaining patients, 36 had benign pleura on histological analysis (7 normal and 29 inflammatory pleural involvement) while 26 had a malignant pleural disease with variable histological findings (Table 2, Fig. S2 in Supplementary Information). A total of 310 images were available for the study and the 11 criteria analysis. Approximately 5 min were necessary after intravenous fluorescein injection for an optimal pleural staining.

**Table 2:** Patients characteristics and histological diagnosis (*n* = 62)

Sex	19 F/43 M
Mean age (SD)	64.4 (17.29)
Thoracoscopy indications	Talc pleurodesis for recurrent pneumothorax: *n* = 8
	Pleural exudation management (mainly lymphocytic): *n* = 46
	Talc pleurodesis for recurrent malignant effusion: *n* = 4
	Pleural hypermetabolism on PET: *n* = 2
	Pleural nodularity on CT scan: *n* = 2
Histological diagnosis	Normal pleura: *n* = 7
	(Sub‐)acute pleuritis: *n* = 15
	Chronic pleuritis: *n* = 13
	Pulmonary adenocarcinoma: *n* = 9
	Malignant mesothelioma: *n* = 7
	Large cell lymphoma: *n* = 2
	Small cell lung carcinoma: *n* = 3
	Squamous cell lung carcinoma: *n* = 3
	Breast adenocarcinoma: *n* = 1
	Urothelial carcinoma: *n* = 1
	Sarcoidosis: *n* = 1

CT, computed tomography; PET, positon emission tomography.

Concerning the feasibility of pCLE during medical thoracoscopy, the Alveoflex probe fitted easily through the working channel of our thoracoscopes and the parietal pleura was easy to reach. However, based on thoracoscopists judgement, the quality of the acquisition was inconstant from one patient to another. Indeed, 20 pCLE were of good quality, 22 of acceptable quality and 20 of low quality.

There were no adverse events related to the pCLE procedure or to the intravenous fluorescein administration.

### Pleural pCLE descriptions

**Figure 1:**
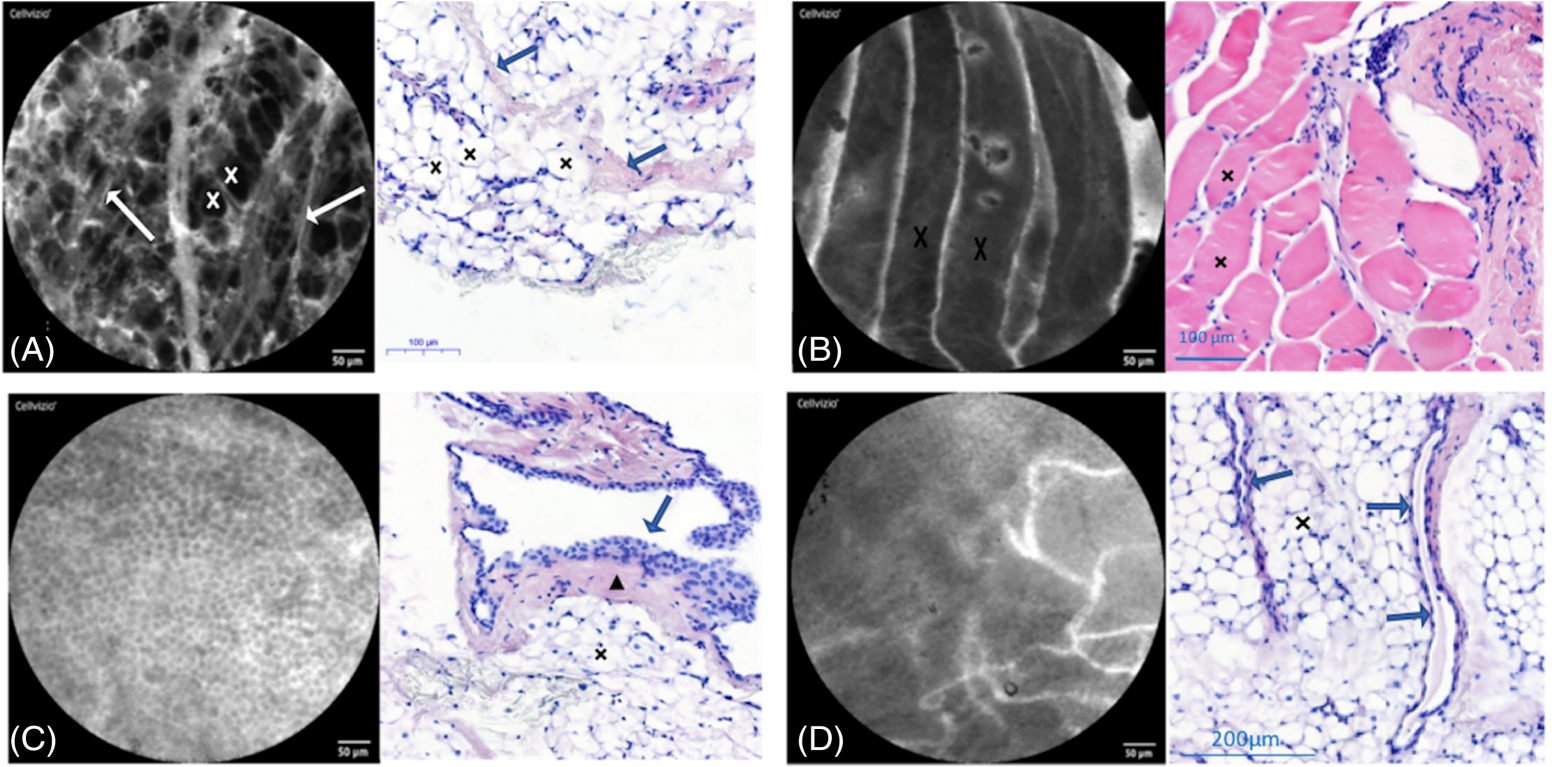
Normal probe‐based confocal laser endomicroscopy (pCLE) features of the parietal pleura (see the pleural pCLE description in the Results section for more details). (A) The pCLE image and the histological section (haematoxylin and eosin (HE)) show fibroadipose connective tissue of the pleura with adipocytes (crosses) and connective fibres (arrows). (B) The histological (HE) section and the pCLE image show striated muscular fibres (crosses). (C) The pCLE image shows the normal mesothelium (‘full chia seed sign’). The histological section (HE) shows a normal pleura. The arrow shows where the pCLE is positioned (perpendicularly to the mesothelial surface) to give the pCLE image. The triangle lies in the sub‐mesothelial connective tissue and the cross highlights the adipocytes (which are not presented on this pCLE image). (D) The pCLE image shows the pleural vascularization and some mesothelial cells. The histological (HE) section shows pleural vessels (arrows) lying in the pleural adipose tissue (cross).

Eight patients were recruited for talc pleurodesis for recurrent spontaneous pneumothorax (with normal or slightly inflammatory pleura on histological section). The normal mesothelium appeared as a well‐organized tissue with polyhedral cells of similar size, shape, fluorescence intensity with well‐delineated intercellular gaps and cell borders. There was a fibroadipose connective tissue beneath the mesothelium featuring well‐delineated large (around 50 μm) round, dark adipocytes surrounded by blood vessels and connective fibres. Sometimes, striated muscular fibres could be imaged as large (100 μm) and long (hundreds of μm) parallel dark bands (Fig. 1, Appendix S2‐Video 1 in Supplementary Information).^13^

**Figure 2:**
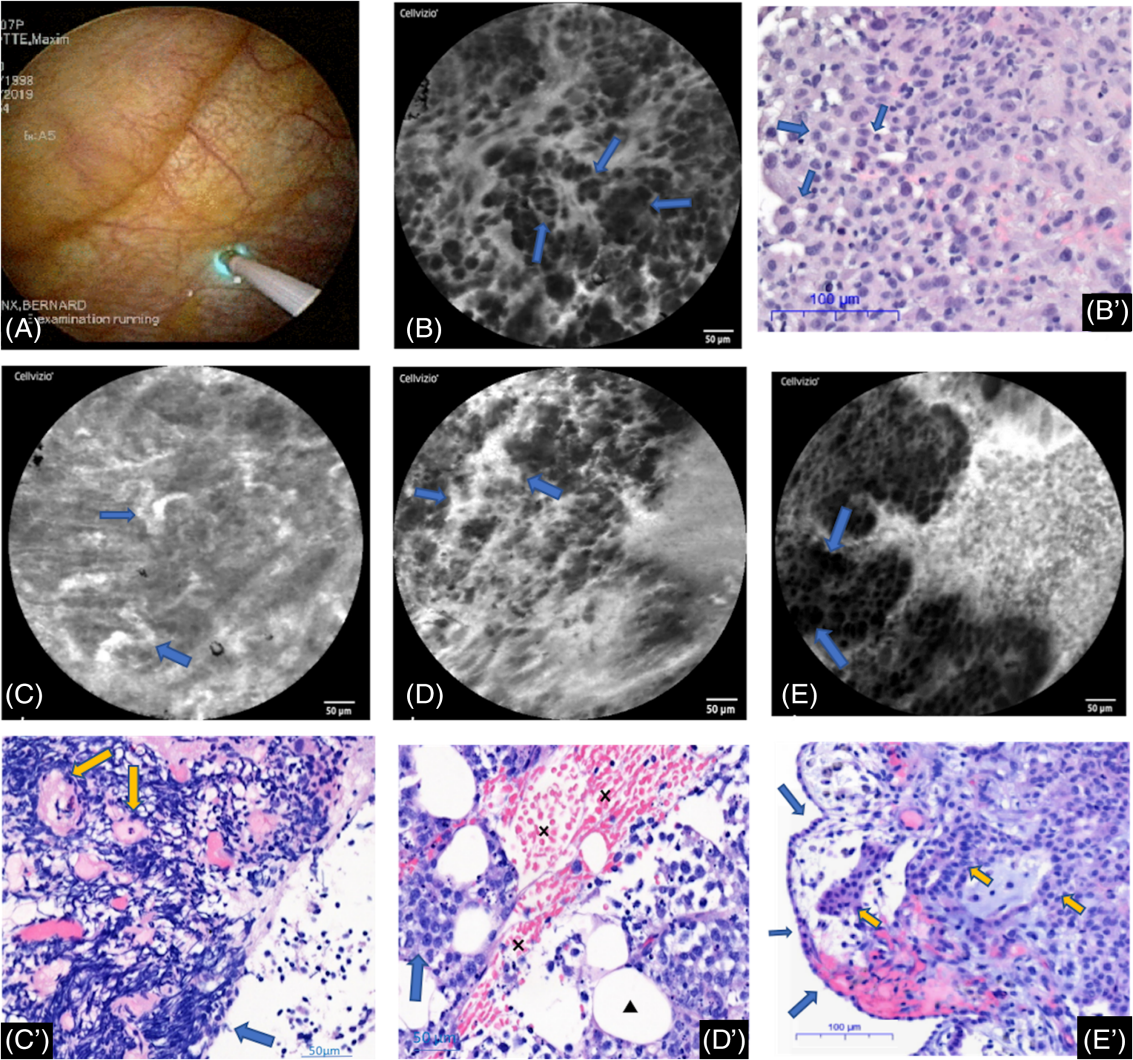
Illustrations of the significant probe‐based confocal laser endomicroscopy (pCLE) features for pleural investigation. (A) pCLE gently applied on the parietal pleura (thoracoscopic view). (B, B′) Abnormal pleural architecture with cells of different size, shape and infiltration by glands (arrows) and cell clusters (lung metastatic adenocarcinoma). Haematoxylin and eosin (HE) staining for the histological section. (C, C′) The pCLE image shows dysplastic tortuous vessels (arrows) in a patient with metastatic lung adenocarcinoma. The histological section (HE) shows tortuous pleural vessels (arrows) within the pleural connective tissue infiltrated by inflammatory cells. (D, D′) The pCLE image shows a dysplastic vessel with patent fluorescein leaks (arrows) in a patient with pleural infiltration by a large B cell lymphoma. The histological section (HE) shows a haemorrhagic suffusion (crosses) in the pleural connective tissue (triangle for adipocytes) infiltrated by malignant B cells (arrow). (E, E′) The histological section (HE) shows a pleura (blue arrows are for the mesothelial layer) infiltrated by malignant cells taking an epithelioid architecture (epithelioid mesothelioma) (yellow arrows). The pCLE image highlights the epithelioid (aberrant) architecture and identifies some cell clusters (arrows). The full chia seed sign is illustrated in Figure 1 with the normal pleural pCLE features.

Twenty‐six patients had malignant pleural diseases. With pCLE, the normal pleura was no more visible and severe architecture and tissue distortions were identified. Indeed, the malignant pleural involvement was characterized by the presence of heterogeneous and pleomorphic cells with distorted intercellular gaps and indistinct cell borders. Those cells could be organized in clusters or nodules or take a papillar, a glandular or another aberrant tissue architecture (Fig. 2B,E, Appendix S2‐Video 2 in Supplementary Information). Sometimes, vascular abnormalities were also identified with vascular leaks, tortuous or giant vessels (Fig. 2C,D).

### Criteria assessment

Among the 11 assessed criteria, four were statistically significantly associated with the malignity or the benignity of the pleural involvement (Tables 3, 4, 5, Fig. 2).

**Table 3:** Qualitative criteria for pleural pCLE assessment

	Benign pleura, *n* = 36			Malignant pleura, *n* = 26			Total not assessable	Fisher's exact test
	Yes	No	N.A.	Yes	No	N.A.	N.A. (%)	*P*‐value
Abnormal architecture	8	21	7	24	0	2	9 (14.5%)	**<0.0001**
Cellular size homogeneity	18	14	4	7	16	3	7 (11.3%)	0.0987
Cellular shape homogeneity	18	14	4	4	19	3	7 (11.3%)	**0.0052**
Cellular fluorescence homogeneity	28	7	1	14	10	2	3 (4.83%)	0.0863
Blood vessels dysplasia	8	23	5	17	3	6	11 (17.7%)	**<0.0001**
Organized connective tissue	11	20	5	5	12	9	14 (22.6%)	0.76
Chia seeds sign	13	23	0	0	26	0	0 (0%)	**0.0003**

N.A., not assessable; pCLE, probe‐based confocal laser endomicroscopy. *p* < 0.05 is significant in our statistical analysis.

**Table 4:** Quantitative criteria for pleural pCLE assessment

	Benign, mean (SD)	Malignant, mean (SD)	N.A. (%)	*P*‐value
Mean cell size (μm)	21.76 (5.69)	22.96 (5.16)	4 (6%)	0.4352
Mean cell density (/10^4^ μm^2^)	25.42 (8.73)	22.01 (7.35)	4 (6%)	0.1404
Maximum vascular diameter (μm)	22.72 (9.93)	27.08 (8.91)	8 (12.9%)	0.1216
Number of blood vessels (/field)	9.81 (7.73)	6.90 (2.53)	11 (17.7%)	0.1223

N.A., not assessable; pCLE, probe‐based confocal laser endomicroscopy.

**Table 5:** Pleural pCLE significant criteria

Criteria	Sensitivity (%)	Specificity (%)	Positive pred value (%)	Negative pred value (%)
Full chia seed sign	36	100	100	53.06
Cell shape homogeneity	56.25	69.57	72	53.33
Dysplastic vessels	85	74	68	88.46
Abnormal tissue architecture	100	72.41	75	100

Abnormal tissue architecture, associated with malignity; cellular shape homogeneity, associated with benignity; dysplastic vessels, associated with malignity; full chia seed sign, associated with benignity; pCLE, probe‐based confocal laser endomicroscopy; pred, predictive.

The identification of the abnormal tissue architecture was significantly associated with malignant pleural involvement (*P* < 0.0001). It was present for all malignant diseases with a good specificity (sensitivity (ss): 100%, specificity (sp): 72.41%, positive predicted value (PPV): 75%, negative predicted value (NPV): 100%).

The ‘full chia seed sign’, which is a sign of normal architecture, had a relatively low sensitivity for benignity but was 100% specific, yielding a 100% PPV (ss: 36%, sp: 100%, PPV: 100%, NPV: 53.06%, *P* = 0.0003). Likewise, the presence of cells homogeneously shaped was associated with benignity but with relatively low sensitivity and specificity (ss: 56.%, sp: 70%, PPV: 72%, NPV: 53%, *P* = 0.0052).

Dysplastic vessels were significantly and strongly associated with malignancy (*P* < 0.0001) with a good sensitivity and specificity (ss: 85%, sp: 74%, PPV: 68%, NPV: 88.46%).

The other assessed criteria (cellular size and fluorescence homogeneity, cellular mean size and density, vascular density and maximum diameter and connective tissue fibre organization) did not show significant association with the final histological diagnosis (Tables 3, 4).

## DISCUSSION

Only two studies concerning pleural diseases pCLE investigation have been published so far and our trial is the largest to assess pCLE during medical thoracoscopy. Normal and malignant pleural pCLE features are precisely described. Furthermore, 11 criteria were selected according to our previous experience^13^ and four of them proved to be associated with the final histological diagnosis (tissue architecture, dysplastic vessels, full chia seed sign and cellular shape homogeneity).

As less invasive pleural investigation procedures such as thoracentesis or Abram's needle biopsies lack sensitivity (less than 60% for repeated thoracentesis),^14^, ^15^ thoracoscopy remains the gold standard for pleural diseases management.^15^, ^16^, ^17^ Therefore, we chose the most appropriate procedure to study and validate the pleural pCLE features with their strictly corresponding histological samples, which is the strength of our work. In fact, the clinical value of pCLE with fluorescein during thoracoscopy remains to be determined, but we believe that pCLE could improve the diagnostic yield of less invasive pleural procedures (such as thoracentesis or Abram's needle biopsies) now that we have precisely described and validated the normal and malignant pleural pCLE features.^8^, ^18^ This should be the third step of our research.

For our study, we used the Alveoflex probe. Because of a confocal plane beginning at 0 μm (standing between 0 and 50 μm), this probe is probably the most appropriate to study the pleural mesothelium, the most superficial layer of the pleura and associated malignancies (e.g. the AQ‐flex, Cellvizio; Maunakea Technologies Paris, France, probe has a confocal plane standing between 40 and 70 μm deep). In our study, the presence of the full chia seed sign is highly specific for benignity with a 100% PPV. In fact, the mesothelium is a really thin, delicate and highly reactive monocellular layer.^19^ Whenever involved in a pathological process, alterations or distortions of the mesothelium can prevent its identification during endomicroscopy. This explains the relatively low sensitivity of this criterion but, also its very high specificity for benignity. Similarly, the cellular shape homogeneity was significantly associated with benignity, although, in this case, with lower sensitivity and specificity.

As for the abnormal tissue architecture, we showed in the current study that the presence of pleomorphic cells, dark clumps and cell clusters was found in all pleural malignancies but only in 27% of the benign pleura. Our finding is in keeping with previously reported studies assessing pCLE in thoracic oncology.^5^, ^7^, ^8^, ^20^ Furthermore, as the Alveoflex probe, with an optic area of 1.13 mm^2^, is the probe with the largest field of view, it allows a better assessment of the general tissue architecture than other probes such as the AQ‐flex which has an optical area of only 0.33 mm^2^. Thereby, the global cellular and tissue organization could be analysed allowing for identification of glands, papils, cordons and macronodules of cells in malignant pleura (Fig. 2).

Dysplastic vessels were significantly and strongly associated with malignancy in our study. Fluorescein is a really good vascular staining agent. In fact, it has been used for decades in ophthalmology to stain retinal vessels.^21^ Our finding is not surprising as (neo) angiogenesis is a hallmark of cancer and is identified as a therapeutic target in malignancies management.^22^ The new vessels are often tortuous, irregular (in size and shape), disorganized and excessively leaky.^23^ In endomicroscopy with fluorescein, the vessels appear clearly as hyperfluorescent images and can be easily and precisely studied in a dynamic fashion. This live and dynamic evaluation of tissues at a cellular level is a hallmark of pCLE (conversely to standard histology). In gastroenterology, the pCLE study of the vascular network is already part of the diagnostic management of malignancies, mostly in pancreatic cystic diseases.^24^, ^25^, ^26^, ^27^ As a consequence, we believe that pCLE live imaging with fluorescein is really suitable to study vascular abnormalities (static and dynamic) in thoracic malignancies.

None of the quantitative criteria were shown to be significantly associated with the histological diagnosis. This could be surprising because malignant involvement is frequently associated with hypervascularization, hypercellularity and tissue heterogeneity. However, because of the tissue heterogeneity, it was difficult to take reproducible and precise measurements with only five pictures per patient.

Regarding the qualitative criteria, it is worth noting that those without significant association with the histological diagnosis are the most subjective criteria with probably the lowest reproducibility. Furthermore, there might be no association between the connective tissue organization, the cell fluorescence homogeneity and the histological diagnosis.

Although promising, our results need to be validated prospectively in larger multicentre studies, with assessment of reproducibility of the results. A major limitation in our experience concerns the quality of the acquisition, as 20 out of 62 set of images were scored low by the two thoracoscopist investigators with some criteria not assessable and therefore had to be excluded from the statistical analysis. Although limited in proportion (Tables 3, 4), this could lead to an overestimation of our results. Several reasons can explain this low quality. First, it was sometimes difficult to stabilize the probe and the image acquisition because of the respiratory movements of the patient (depending, notably on the sedation depth), the curved shape of the thorax and the high flexibility of the probe. Second, when present, a pachypleuritis or a thick fibrinous pleuritis could prevent a good visualization of the underlying mesothelial layer or other pleural structures either because of an increased tissue thickness and/or a suboptimal fluorescein pleural staining. However, the quality of the acquisition has gradually improved along the study (learning curve). Therefore, we believe that experienced investigators could reach a more favourable proportion of interpretable pleural pCLE investigation, as already shown by others.^28^, ^29^

Another potential bias of our study is the selection of the ‘most representative images’ by the same investigators than the one who scored the criteria. However, we think that this potential bias is limited for qualitative criteria (the only ones to be significant in our study). In fact, those criteria are binary scored (yes or no) and the scoring investigator screened the entire pCLE acquisition to find the best representative images. As for quantitative criteria, which are not significant, we acknowledge a possible bias because of the high heterogeneity of some acquisitions.

Finally, the criteria investigated in the present study are based on a small number of patients (*n* = 7) recruited for the first step of our investigation. A larger prediction set would have strengthened the study.

In conclusion, this is the first study evaluating pCLE during medical thoracoscopy. Our results demonstrate the safety and the feasibility of the technique. We provide a precise description of benign and malignant pleural pCLE features validated by the gold standard procedure for pleural diseases investigation. Discriminating criteria between malignant and benign pleura were identified. We believe that pCLE for pleural disease investigation and management could be a valuable tool. Whether pCLE could become part of minimally invasive pleural investigation procedure or increase the diagnostic accuracy of thoracentesis needs to be investigated in further studies.

## Author contributions

Conceptualization: O.B., V.H., N.D. Formal analysis: O.B., J.‐L.C., B.D., R.L. Funding acquisition: O.B., V.H. O.B., V.H., B.D., J.‐L.C., R.L. Data curation: O.B., V.H., N.D. Formal analysis: O.B., J.‐L.C., B.D., R.L. Funding acquisition: O.B., V.H. Investigation: O.B., V.H., R.L., N.D., J.‐L.C., B.D. Methodology: O.B. Project administration: O.B., V.H., J.‐L.C., R.L., B.D. Resources: O.B., R.L. Software: O.B. Supervision: O.B., V.H., J.‐L.C., R.L., N.D., B.D. Validation: O.B., J.‐L.C., R.L., B.D. Writing—original draft: O.B., V.H. Writing—review and editing: V.H., J.‐L.C., R.L., N.D., B.D.

## Supporting information


**Appendix** **S1** Preselected criteria for pCLE pleural evaluation.
**Appendix S2** Video descriptions.
**Video 1:** Normal pleura.
**Video 2:** Epithelioid mesothelioma
**Figure S1** Illustration of the chia seed sign.
**Figure S2** Probe‐based confocal laser endomicroscopy for pleural malignancies diagnosis: Flow chart.

## Data Availability

The patients' individual data will not be available.
