# Supplementary material for: Probe‐based confocal laser endomicroscopy for pleural malignancies diagnosis
Source: Respirology. 2020 Oct 1;26(2):188–95. doi: 10.1111/resp.13945 (PMC7891618; doi:10.1111/resp.13945)
Supplement: Supplementary file 1 — Appendix S1 Preselected criteria for pCLE pleural evaluation. Appendix S2 Video descriptions. Video 1: Normal pleura. Video 2: Epithelioid mesothelioma Figure S1 Illustration of the chia seed sign. Figure S2 Probe‐based confocal laser endomicroscopy for pleural malignancies diagnosis: Flow chart. [file RESP-26-188-s001.zip › RESP_13945_RES-20-135.R1-Suppl-Info (4).doc]

**SUPPLEMENTARY INFORMATION**

**Probe based confocal laser endomicroscopy for pleural malignancies diagnosis.**

O. Bonhomme¹ M.D, V. Heinen¹ M.D, N. Detrembleur²M.D, J-L. Corhay¹ M.D, PhD, R. Louis¹ M.D, PhD, B. Duysinx¹ M.D, PhD.

^1^ Pneumology Department, CHU Liège, Liège, Belgium; Domaine universitaire du Sart-Tilman, B35

B4000 Liège Belgium

^2^ Pathology Department, CHU Liège, Liège, Belgium ; Domaine universitaire du Sart-Tilman, B35

B4000 Liège Belgium

**Appendix S1- Preselected criteria for pCLE pleural evaluation**

1. The abnormal tissular architecture was defined by the identification of tissular/cellular structures not known to belong to the normal pleura (cell clusters, dark clumps, glands, cell cordons, dysmorphic cells, papillar architecture, glands…). The absence of abnormal architecture was defined by the identification of the normal pleural cellular and tissular elements as previously reported: the mesothelial monolayer with homogeneous and regularly distributed polyhedral cells; the fibro-adipose connective tissue and its regular and homogeneous vascularization; muscular fibers.
2. The cells homogeneity in size, fluorescence and shape was subjectively assessed by the investigator.
3. The mean cellular size was assessed by measurement of the largest cells diameters on the full optical area of the probe (1.13 mm²).
4. The mean cellular density is obtained by three measurements in different areas of 10.000µm² where the cellular density was visually the highest.
5. The presence of dysplastic vessels was assessed, as described by Cannizzaro et al., by the identification of vascular leaks (fluorescein leakage outside the vessels), tortuous vessels or giant vessels ^14^ in the set of five pCLE images.
6. For the maximal vascular diameter, we selected the largest vessel among the available pCLE images in the set of every patient.
7. For the vascular density, we measured the max number of vessels/image (full optical area) for every patient on the available images. Vessels are defined as continuous tubular hyper fluorescent images of at least 50 µm. The image with the highest vascular density was selected.
8. The connective tissue fibers were classified as anarchic or organized. Anarchic connective tissue fibers were defined as irregular in shape or direction, coarse, without a well-defined architecture. Organized connective tissue fibers were defined as regular in shape and direction with a well-defined architecture.
9. The full chia seed sign defines the presence on a full optical area of the mesothelial monolayer with well defined, homogeneous and organized mesothelial cells (fig 1).

The criteria were scored “not assessable” if elements for their interpretation were missing.

**Appendix S2**-**Video descriptions**

Video 1: normal pleura.

The first part of the video shows the pleural connective tissues with connective fibers and some adipocytes. In the second part, the mesothelium is identified as a well-organized tissue with homogeneously distributed cells of similar size, shape and fluorescence intensity with well delineated cells borders. Some adipocytes can be identified as well-defined round dark and large cells

Video2: epithelioid mesothelioma

The normal pleura elements are no more visible and severe architecture and tissular distortions are identified. Indeed, the video shows the presence of heterogeneous and pleomorphic cells with distorted intercellular gaps and indistinct cell borders. Those cells are organized in clusters or nodules or take a papillar aberrant tissular architecture

**Figure S1 – Illustration of the chia seed sign**

Illustration of the full chia seed sign: illustration of the pleural mesothelial layer during pCLE (left side of the picture) when the probe is gently applied on the parietal pleura. This looks like a bowl of chia seeds in almond milk (right side of the picture), hence the name of this specific pCLE feature: the full chia seed sign.

**Figure S2-Probe based confocal laser endomicroscopy for pleural malignancies diagnosis**

**Flow Chart**

Screened patients

N = 65

One patient refused

Included patients

N = 64

Patients included in final analysis

pCLE during medical thoracoscopy

N = 62

Lack histological conclusion : one patient

pCLE images no more available : one patient

Malignant pleural infiltrations

N = 26

Beningn Pleura

N = 36
